# Effects of Dietary Bile Acids on Growth Performance, Lipid Deposition, and Intestinal Health of Rice Field Eel (*Monopterus albus*) Fed with High-Lipid Diets

**DOI:** 10.1155/2023/3321734

**Published:** 2023-12-27

**Authors:** Wei Lei, Jiamin Li, Peng Fang, Shanshan Wu, Yao Deng, Ao Luo, Zhengwei He, Mo Peng

**Affiliations:** ^1^College of Animal Science and Technology, Jiangxi Agricultural University, Nanchang 330045, China; ^2^Key Laboratory of Featured Hydrobios Nutritional Physiology and Healthy Breeding, Nanchang 330045, China

## Abstract

The purpose of this trial was to study the positive effects of bile acids (BAs) on growth performance and intestinal health of rice field eel fed with high-lipid diets (HLDs). Rice field eels (initial weight 17.00 ± 0.10 g) were divided into four groups, each group containing four repetitions and feeding with different isonitrogenous diet: control diet containing 7% lipid content, HLDs containing the lipid content increased to 13%, HLDs supplementing with 0.025% BAs and 0.05% BAs, respectively. After 8 weeks, compared control group, the fish fed HLDs had no significant effect on weight gain rate and specific growth rate (*P* > 0.05), but increased the lipid deposition in tissues and intestinal lipase activity, and damaged to intestinal oxidative stress, inflammatory response, physical barrier, and structural integrity (*P* < 0.05). Dietary BAs significantly increased weight gain rate and specific growth rate in fish fed with HL diets (*P* < 0.05) and reduced feed conversation rate (*P* < 0.05). Further, the eels fed with BAs reduced the total lipid content in liver, muscle, and whole body (*P* < 0.05). Dietary BAs decreased the activity of intestinal lipase (*P* < 0.05). Meanwhile, BAs supplemented in HLDs improved intestinal antioxidant capacity through increasing the activities of T-SOD (total superoxide dismutase), GSH-PX (glutathione peroxidase), CAT (catalase), T-AOC (total antioxidant capacity), whereas reducing MDA (malondialdehyde) content (*P* < 0.05). Moreover, dietary BAs regulated the mRNA expression related to inflammatory response, oxidative stress, and physical barrier in intestine, such as *tnf-α*, *il-8*, *tlr-8*, *il-10*, *nrf2*, *keap1*, *claudin12*, and *claudin15* (*P* < 0.05). Dietary BAs supplementation also enhanced the intestinal structural integrity characterized by increased fold height and lamina propria width (*P* < 0.05). This study showed that dietary BAs supplemented in HLDs (13% lipid) could increase the growth performance of rice field eel, reduce lipid deposition in tissues and whole body, and enhance intestinal health.

## 1. Introduction

With the rapid development and scale expansion of aquaculture, the requirement of superior protein sources, especially fishmeal, is increasing. As a result, the feed cost was maintained high level [1]. High-lipid diets (HLDs) could provide more energy and show protein-sparing effect to reduce cost in aquaculture [2–8]. However, HLDs often induce undesirable effects on lipid deposition and health of liver and intestine, then decrease the growth and feed utilization in grass carp (*Ctenopharyngodon idella*) [9, 10], cuneate drum (*Nibeamiichthioides*) [11], and Chinese perch (*Siniperca chuatsi*) [12]. Especially, HLDs triggered adverse effects on intestinal digestion and antioxidant capability [13–16]. To address the dual effects of HLDs, effective methods are needed to mitigate their harmful impact on growth performance and intestinal function in farmed fish.

Bile acids (BAs) are a natural emulsifier and *de novo* biosynthesized from cholesterol in the liver. They can regulate metabolism and health via accelerating lipid absorption and transportation and activating nuclear receptors [17]. Previous studies in pigs proved that exogenous BAs could increase growth performance, regulate lipid metabolism, and improve health [18, 19]. Therefore, application of exogenous BAs on farmed fish has received widespread attention. Studies on Chinese perch [20] and tongue sole (*Cynoglossus broadhursti*) [21] indicated that BAs supplemented in the normal diet could improve growth performance, as well as the antioxidant capacity in intestine. Similarly, studies on other fish have shown that dietary BAs increased growth performance and alleviated liver and intestinal dysfunction caused by high plant protein or high carbohydrate diets [22–25].

Dietary BAs presented positive effects in numerous fish fed with HLDs. Dietary BAs increased the growth performance in large yellow croaker (*Larimichthys crocea*) [26] and grass carp [27], reduced hepatic lipid deposition, improved liver antioxidant capacity, or mitigated endoplasmic reticulum stress in largemouth bass (*Micropterus salmoides*) [28], hybrid grouper (*Epinephelus lanceolatus♂× E. fuscoguttatus♀*) [29], large yellow croaker [30], and tiger puffer (*Takifugu rubripes*) [31] fed with HLDs. Dietary BAs also altered the intestinal microbiota composition and BAs profiles in grass carp and hybrid grouper [27, 29], or increase intestinal mucosa in largemouth bass [28]. However, whether or not dietary BAs could improve intestinal health in fish fed with HLDs still lacks of evidence. Furthermore, the intestinal function is vital for fish growth [32]. It is worthy to systematically evaluate the effects of exogenous BAs on intestinal function of farmed fish consuming HLDs.

Rice field eel is a famous cultured carnivore fish in China for its delicious and delicate meat and nutritional values. The fish is sensitive to dietary lipid and carbohydrate levels in diets [15, 33, 34]. Previous studies found that HLDs decreased the growth, increased liver lipid deposition, undermined the homeostasis of intestinal microbiota, and caused to intestinal dysfunction [33–35]. Moreover, the positive effects of functional additives on eel fed different nutritional diets were proven recently [36–39]. It is reasonable to further study the effect of another functional additive on the growth and intestinal health of this fish continuously. Thus, the aim of the present experiment is to explore effects of dietary BAs supplementing in HLDs on the growth performance and intestinal health of rice field eels. After that, results would provide a nutritional strategy to mitigate the adverse effect of HLDs and then increase the usage of HLDs and exogenous BAs in freshwater aquatic animals.

## 2. Materials and Methods

### 2.1. Diets Preparation

Fishmeal and wheat gluten were used as the main protein source in this trial, fish oil and soybean oil were used as the main lipid source, and the main sugar source was wheat starch. Four isonitrogenous diets (the percentage of protein ranged from 42% to 43%) were formulated as follows (Table 1), a diet formulated containing 7% crude lipid was denoted as control (CON), a diet formulated containing approximately 13% crude lipid was denoted as HLDs (HL), the HL diet supplemented with 0.025% BAs (HLLB) and 0.05% BAs (HLHB) were adopted to investigate the effect of dietary BAs on fish. All ingredients were crushed and passed through 60 mesh sieve, and then fish oil and soybean oil were equally added and thoroughly mixed, the final product material was also powder. Before using, the diets were stored at −20°C. Placing the diets at room temperature for 1 hr before daily feeding, and added 20% water into the diets to make ball-shaped dough [40].

### 2.2. Experimental Animal and Feeding Experiment

Fish were bought from the eel farm in Yugan, Jiangxi, China. All individuals were bred in floating cages (2.0 m × 2.0 m × 1.5 m) and fed for 2 weeks to improve the adaptability to the water environment and the experimental diets. Selected 960 healthy eels weighing 17.10 ± 0.1 g were randomly distributed into 16 float cages (2.0 m × 2.0 m × 1.5 m) after fasting for 24 hr. Each group contained four cages, 60 fish per cage. During the 56-day breeding trial, the fish were artificially fed once a day (5:00 pm–6:30 pm) in apparent satiety.

### 2.3. Sampling

After the eels were starved for 24 hr, anaesthetized the eels with 100 mg/L MS222, weighed and counted the fish in each cage. Measured and weighed fish body weight and length, visceral mass, and liver weight separately to calculate the indices of growth performance. Portions of intestine, liver, and muscle of three fish were got out and placed in a 1.5 mL test tube, for determining antioxidants, immune parameters, and lipid deposition in tissues. The above samples were preserved at −80°C. Three fish were taken from each cage and then stored frozen at −20°C for analysis of the chemical composition in the whole body. The middle sections of the intestine sample were placed and filled in 4% paraformaldehyde for histological analysis.

The survival rate (SR), weight gain rate (WGR), specific growth rate (SGR), feed conversion rate (FCR), condition factor (CF), hepatosomatic index (HSI), and viscerosomatic index (VSI) were calculated according to previous study in this eel [41].

### 2.4. Chemical Composition Analysis

The experimental diets and the fish were oven-dried at 105°C in the oven to calculate the moisture content. The crude protein content in diet and fish was determined by Kjeldahl method and the crude lipids in diets and the fish were determined using a Soxhlet extraction method. Ash in diets was estimated after being burned at 550°C in a muffle furnace [31]. The above-detailed methods were seen in standard laboratory procedures [42]. The liver and muscle total lipid contents were determined by methanol–chloroform (1 : 2) method [43].

### 2.5. Histological Analysis

The midgut of rice field eel (5 mm × 5 mm) was fixed in 4% paraformaldehyde solution for 24 hr. After that, dehydrated with alcohol and washed with xylene. Then embedded in paraffin wax, cut sections with 5 *μ*m thickness, and stained with hematoxylin and eosin (H&E). This method was adapted from previous study and adjusted [41]. Histological results were analyzed by using Olympus BX53.

### 2.6. Biochemical Analysis

The activities of intestinal digestive enzymes (trypsin, lipase, and amylase), indices related to intestinal antioxidant capability, glutathione peroxidase (GPX, NO: A005-1-2), malondialdehyde (MDA, NO: A003-1-2), total antioxidant capacity (T-AOC, NO: A015-2-1), total superoxide dismutase (T-SOD, NO: A001-3-2), and catalase (CAT, NO: A007-1-1), were analyzed through the commercial kits (Nanjing Jiancheng Biotechnic Institute, China). Simply, the intestine was homogenized to obtain the supernatant, and the total protein (TP, NO: A045-4-2) content was measured for error calibration. Subsequently, the supernatant and reagents were combined and used for measuring the abovementioned parameters using a microplate reader or colorimetry methods.

### 2.7. RT-PCR

Total RNA was extracted from the intestine, and the integrity was tested by RNA electropherogram. Afterwards, using the Prime ScriptTM kit (Takara, Japan) to reverse transcription of the extracted total RNA into cDNA. The qRT-PCR was performed by Mastercycler ep realplex (Eppendorf, Germany), and reaction volume was 10 *μ*L (primers (0.4 *μ*L), cDNA (0.5 *μ*L), SYBR Green qPCR Mix (High ROX) (Aidlab Biotechnologies Co. Ltd., China) (5 *μ*L), and sterile nonenzyme water (4.1 *μ*L)). The qPCR program was set as 95°C for 2 min, followed by 40 cycles of 95°C for 15 s, *T*m for 30 s, and 72°C for 30 s. Adopting the 2^−*ΔΔ*Ct^ method to calculate the genes expression [44]. According to the result of amplification efficiency, then selected 18s ribosome RNA as the internal reference gene of this study. The primer sequences are shown in Table 2.

### 2.8. Statistical Analysis

In this experiment, SPSS software 25.0 (SPSS Inc., USA) was used to test the normality and homoscedasticity of the data. The differences between the CON and HL groups were analyzed using Student's *t*-test, while the differences among the HL, HLLB, and HLHB groups were analyzed by one-way ANOVA and Duncan multiple range tests. Meanwhile, the effect of BAs was linear or not was determined by using orthogonal polynomial contrast. All data are represented as mean ± SEM (standard error of mean), *P* < 0.05 is set as statistically significant. All raw data were handled by Microsoft Excel 2017 (Microsoft, USA), and then drafted them into three-line tables by Microsoft Word 2017 (Microsoft, USA) and figures by GraphPad Prism 5 (GraphPad Software, USA), respectively.

## 3. Results

### 3.1. Growth Performance

HLDs did not affect FBW, WGR, SGR, and HSI (*P* > 0.05), but significantly affected the VSI and decreased the FCR and CF (*P* < 0.05) (Table 3). The WGR, FBW, and CF in HL group were significantly lower than in the two dietary BAs treated groups (*P* < 0.05). FCR in HL group was significantly higher than that in the HLLB group (*P* < 0.05) but was not different with that in HLHB group (*P* > 0.05). Furthermore, the WGR, FBW, and SGR in HLLB group were significantly higher than that in HLHB group (*P* > 0.05). Meanwhile, only the WGR, FBW, FCR, and CF were affected by dietary BAs in significantly linear trend (*P* < 0.05).

### 3.2. Chemical Composition in Whole Body and Lipid Content in Liver and Muscle

**Figure 1:**
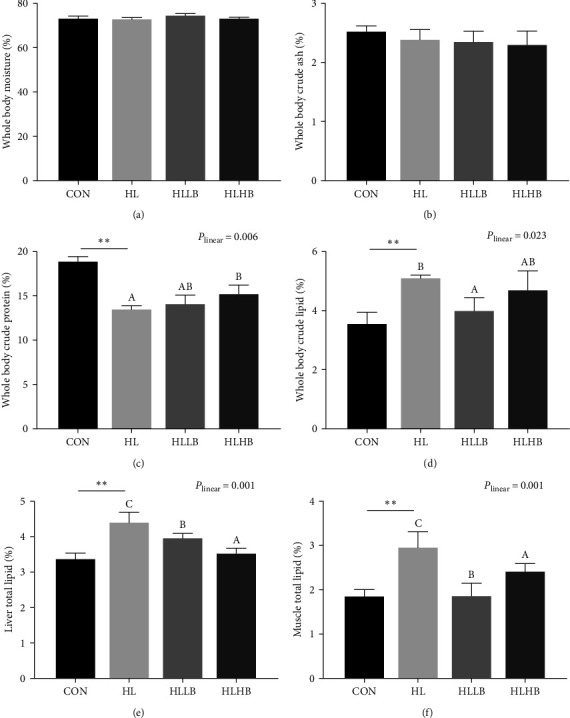
Effects of dietary bile acids (BAs) on the body composition and total lipid content in liver and muscle of rice field eel (*Monopterus albus*) fed with HLDs. Each data point represents the mean of four replicates (*n* = 4, mean ± SEM).  ^*∗∗*^Means *P* < 0.01 between the CON and the HL group. Bars assigned with different superscripts are significantly different (*P* < 0.05) among the HL, HLLB, and HLHB groups. *P*_linear_ means statistical results of orthogonal polynomial contrasts. ns, no significant. Whole body moisture (a), whole body ash (b), whole body crude protein (c), whole body crude lipid (d), liver total lipid (e), and muscle total lipid (f). Treatment groups: CON, control group; HL, high lipid diets group; HLLB, high lipid diets supplementing with 0.025% bile acid group; and HLHB, high lipid diets supplementing with 0.05% bile acid group.

HLDs significantly increased total lipid content in whole body, liver, and muscle (*P* < 0.05), decreased the crude protein in whole body (*P* < 0.05), but did not affect the moisture and ash in whole body (*P* > 0.05) (Figure 1). Compared to HL group, the total lipid contents of liver and muscle were significantly decreased in the two dietary BAs groups (*P* < 0.05). However, the crude lipid content of whole body in HL group was only significantly lower than in lower dietary BAs group (*P* < 0.05). The crude protein content in HL group was only significantly lower than in higher dietary BAs group (*P* < 0.05). Dietary BAs unaffected moisture and ash content in whole body among the three groups (*P* > 0.05). Meanwhile, total lipid contents in liver, muscle, and whole body were decreased and crude protein in whole body increased by dietary BAs in a significant linear trend (*P* < 0.05).

### 3.3. Intestinal Digestive Enzyme Activities

**Figure 2:**
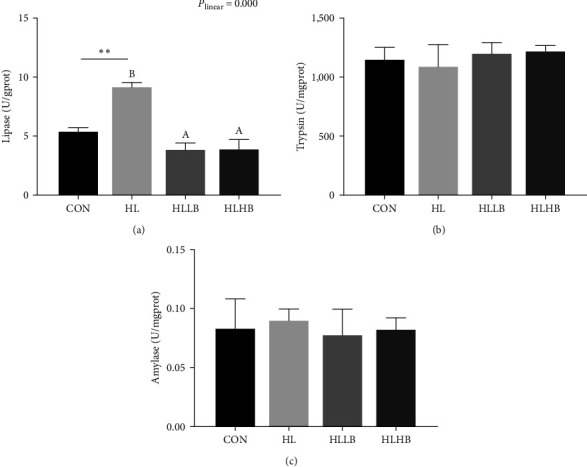
Effects of dietary bile acids (BAs) on the intestinal digestive capacity of rice field eel (*Monopterus albus*) fed with HLDs. Each data point represents the mean of four replicates (*n* = 4, mean ± SEM).  ^*∗∗*^Means *P* < 0.01 between the CON and the HL group. Bars assigned with different superscripts are significantly different (*P* < 0.05) among the HL, HLLB, and HLHB groups. *P*_linear_ means statistical results of orthogonal polynomial contrasts. Intestinal digestive enzyme: lipase (a), trypsin (b), and amylase (c) activities. Treatment groups: CON, control group; HL, high lipid diets group; HLLB, high lipid diets supplementing with 0.025% bile acid group; and HLHB, high lipid diets supplementing with 0.05% bile acid group.

For intestinal digestive enzymes, HLDs significantly increased lipase activity (*P* < 0.05), but did not influence trypsin and amylase activities (*P* > 0.05) (Figure 2). The lipase activity in HL group was significantly higher than that in the two dietary BAs treated groups (*P* < 0.05), but the amylase and trypsin activities among all groups were comparable (*P* > 0.05). Meanwhile, only the lipase activity indicated a significant reducing linear trend in the three HLDs feeding groups (*P* < 0.05).

### 3.4. Intestinal Antioxidant Capability

**Figure 3:**
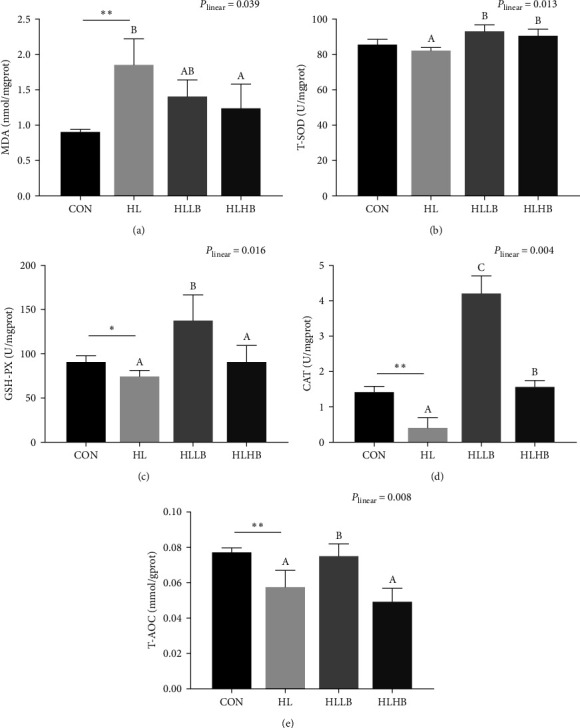
Effects of dietary bile acids (BAs) on the intestinal antioxidant capacity of rice field eel (*Monopterus albus*) fed with HLDs. Each data point represents the mean of four replicates (*n* = 4, mean ± SEM).  ^*∗*^Means *P* < 0.05 between the CON and the HL group,  ^*∗∗*^means *P* < 0.01. Bars assigned with different superscripts are significantly different (*P* < 0.05) among the HL, HLLB, and HLHB groups. *P*_linear_ means statistical results of orthogonal polynomial contrasts. Intestinal antioxidant enzyme: MDA (a), T-SOD (b), GSH-PX (c), CAT (d), and T-AOC (e) activities. MDA, malondialdehyde; T-SOD, total superoxide dismutase; GSH-PX, glutathione peroxidase; CAT, catalase; and T-AOC, total antioxidant capacity. Treatment groups: CON, control group; HL, high lipid diets group; HLLB, high lipid diets supplementing with 0.025% bile acid group; and HLHB, high lipid diets supplementing with 0.05% bile acid group.

HLDs significantly increased MDA content and reduced the activities of CAT, GSH-PX, and T-AOC in intestine (*P* < 0.05) but did not affect T-SOD activity (*P* > 0.05) (Figure 3). The T-SOD and CAT activities in the two dietary BAs treated groups, GSH-PX and T-AOC in HLLB group, were significantly improved than those in HL group (*P* < 0.05). However, the content of MDA in HLHB group was significantly decreased than in HL group (*P* < 0.05). In addition, activities of GSH-PX, CAT, and T-AOC in HLLB group were significantly higher than those in HLHB group (*P* < 0.05). Furthermore, all of the indices relative to intestinal antioxidant capability were affected by dietary BAs in a significant linear trend (*P* < 0.05).

### 3.5. Expression of Genes Related to the Intestinal Health

**Figure 4:**
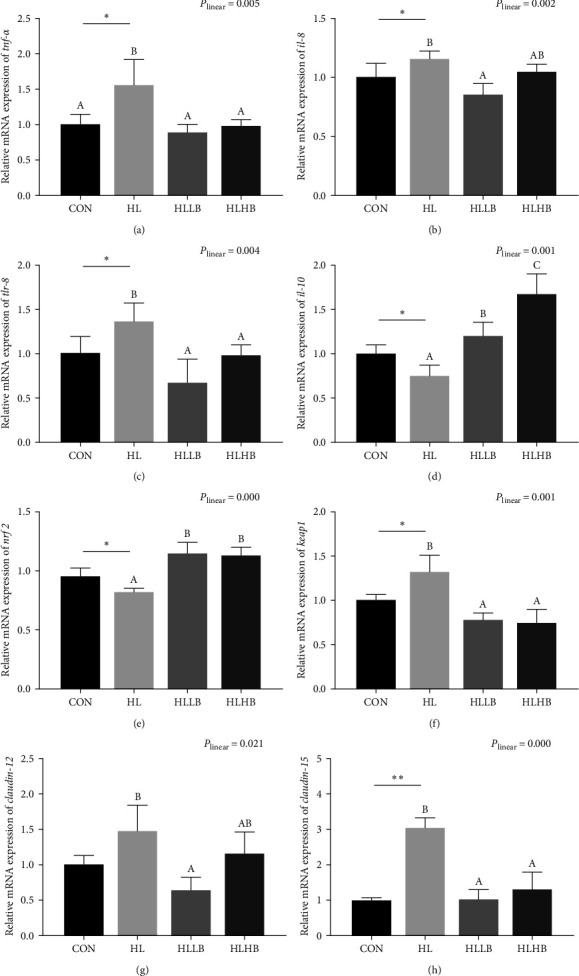
Effects of dietary bile acids (BAs) on the mRNA expression related to the intestinal health of rice field eels (*Monopterus albus*) fed with HLDs. Each data point represents the mean of four replicates (*n* = 4, mean ± SEM).  ^*∗*^Means *P* < 0.05 between the CON and the HL groups,  ^*∗∗*^means *P* < 0.01. Bars assigned with different superscripts are significantly different (*P* < 0.05) among the HL, HLLB, and HLHB groups. *P*_linear_ means statistical results of orthogonal polynomial contrasts. Intestinal pro-inflammatory factors: *tnf-α*, *il-8*, and *tlr-8* (a–c), anti-inflammatory factor: *il-10* (d), oxidative stress-related genes: *nrf2* and *keap1* (e, f), intestinal immune barrier-related factors: *claudin12* and *claudin15* (g, h). *tnf-α*, tumor necrosis factor alpha; *il-8*, interleukin-8; *tlr-8*, toll-like receptor 8; *il-10*, interleukin-8; *nrf2*, nuclear factor erythroid 2-related factor 2; *keapl*, kelch-like ECH associating protein 1; *claudin12*, CLDN12; *claudin15*, CLDN15. Treatment groups: CON, control group; HL, high lipid diets group; HLLB, high lipid diets supplementing with 0.025% bile acid group; and HLHB, high lipid diets supplementing with 0.05% bile acid group.

In Figure 4, HLDs significantly upregulated mRNA expression levels of genes related to inflammatory response (*tnf-α*, *il-8*, and *tlr-8*), oxidative stress (*keap1*), immune barrier (*claudin12* and *claudin15*) (*P* < 0.05) but downregulated mRNA expression levels of *il-10* and *nrf2* (*P* < 0.05). Compared to HL group, mRNA expression levels of *tnf-α*, *tlr-8*, *keap1*, and *claudin15* in both dietary BAs treated groups, and *il-8* and *claudin12* in HLLB group, were remarkably downregulated (*P* < 0.05). However, mRNA expression levels of *il-10* and *nrf2* in both dietary BAs treated groups were significantly upregulated than in HL group (*P* < 0.05). Meanwhile, all the genes expressions relative to intestinal health were affected by dietary BAs in a significant linear trend (*P* < 0.05).

### 3.6. Histological Analysis of Intestinal Morphology

**Figure 5:**
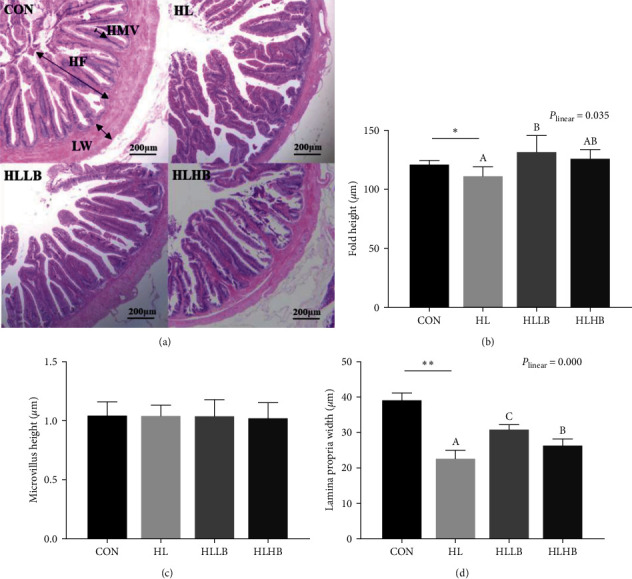
Effects of dietary bile acids (BAs) on the intestinal morphology of rice field eels (*Monopterus albus*) fed with HLDs. Each data point represents the mean of four replicates (*n* = 4, mean ± SEM).  ^*∗*^Means *P* < 0.05 between the CON and the HL groups,  ^*∗∗*^means *P* < 0.01. Bars assigned with different superscripts are significantly different (*P* < 0.05) among the HL, HLLB, and HLHB groups. *P*_linear_ means statistical results of orthogonal polynomial contrasts, (a) Histological results of intestine by hematoxylin and eosin staining. Original magnification is 40x. HF, fold height (b); HMV, microvilli height (c); and LW, lamina propria width (d). Treatment groups: CON, control group; HL, high lipid diets group; HLLB, high lipid diets supplementing with 0.025% bile acid group; and HLHB, high lipid diets supplementing with 0.05% bile acid group.

Histology of gut morphology was observed in Figure 5. The HL group exhibited a significantly lower fold height and lamina propria width than those in CON group (*P* < 0.05). However, microvilli height was similar between the HL and CON groups (*P* > 0.05). The value of lamina propria width in HLLB and HLHB groups was higher than in HL group (*P* < 0.05), but only the fold height in HLLB group was higher than in HL group (*P* < 0.05). Meanwhile, the fold height and lamina propria width were increased by dietary BAs in a significant linear trend (*P* < 0.05).

## 4. Discussion

The present study showed that HLDs could not decrease WGR in this eel. The result was similar to studies in white seabream (*Diplodus sargus*) [45], nile tilapia (*Oreochromis niloticus*) [46], grass carp [47], and black seabream (*Acanthopagrus schlegelii*) [48]. HLDs also caused to adverse effect on growth performance in black sea bream [49], black seabream [48], and Chinese perch [12]. The discrepancy suggested that effects of HLDs on growth performance in farmed fish were complicated. However, HLDs increased the VSI, lipid deposition in liver, muscle, and whole body in this trial. The negative effects of HLDs on lipid deposition in tissues or whole body were found in black seabream [50], grass carp [47], Nile tilapia [46], and turbot (*Scophthalmus maximus* L.) [51], and other farmed fish characterized by positive growth performance [29, 52]. Furthermore, this eel and other fish fed with HLDs often showed lower crude protein content in tissues or whole body [26, 53]. Therefore, it is need to search for effective functional factor to mitigate the adverse effect of HLDs on growth performance and lipid deposition in farmed fish.

Exogenous BAs was used as functional additives to improve growth performance and show lipid-lowering effect in farmed fish [1]. The main objective of this trial was to evaluate the positive effect of dietary BAs on weight gain and lipid deposition. Interestingly, in this study, dietary BAs not only increased the WGR and SGR of this eel but also reduced lipid deposition in liver, muscle, and whole body. Combined with similar results in Chinese perch and large yellow croaker fed with HLDs [1, 29], it can be concluded that supplementing exogenous BAs into diets is an effective approach to regulate growth and lipid metabolism of farmed fish fed with HLDs. Furthermore, it is worth noting that dietary BAs reduced the FCR in this trial, which is similar to the results in snakehead (*Channa* argu*s*) [54] and European seabass (*Dicentrarchus labrax*) [2]. Exogenous BAs also showed positive effect on feed utilization in farmed fish.

The normal intestinal function is thought to be vital for fish health and sustainable aquaculture [32]. HLDs displayed side effects on the intestinal function in farmed fish and then damaged the growth performance [55, 56]. Another important purpose of this study was to evaluate the impact of functional BAs on improving intestinal function.

Lipid peroxidation and proliferation of reactive oxygen species (ROS) were generally produced by feeding fish with long-term HLDs [47, 57]. MDA is the biomarker of lipid peroxidation [58]. To increase ROS removal, several antioxidant response pathways are activated [59, 60]. In the process, antioxidant enzymes are secreted and play an important role [61, 62]. This study illustrated that dietary BAs increased the intestinal activities of T-SOD, GSH-PX, and CAT, whereas reduced MDA content. As a consequence, dietary BAs increased the T-AOC activity. The favorable effects of BAs on antioxidant capability were also found in liver of other fish fed with high lipid or carbohydrate diets [23, 26, 28], and in intestine of Chinese perch fed with normal diets [20], but relative studies in intestine were still rare. The positive impact of BAs on fighting for intestinal oxidative stress needs further exploration on different farmed fish fed with HLDs. In order to explore the molecular mechanism, the Nrf2-Keap1 pathway at gene level was measured. The results indicated that BAs positively regulated Nrf2-Keap1 pathway at genes level. These studies in fish were in line with results in mammals and commercial pigs [63, 64] can conclude that the antioxidant action of BAs may be dependent on the MDA scavenging and antioxidant response boosting through regulating the Nrf2-Keap1 pathway.

As further evidence, dietary BAs demonstrated active impact on relieving inflammatory reaction in intestine induced by soybean oil diets in large yellow croaker [30, 65]. The nutritional background was different between large yellow croaker and this eel, but effects of BAs directly suppressing the gene expression of pro-inflammatory factor and upregulating anti-inflammatory factor were similar. In addition, supplementation exogenous BAs attenuated the inhibition of gene expression relative to cytokines of inflammatory response and protein expression of NF-*κ*B in grass carp fed with low-protein/HLDs [8]. Furthermore, TLR-2 immunoreactivity in gut of goldfish was activated by a high-cholesterol diet [66], and HLDs could activate the TLR-NF-*κ*B pathway and then result in *tnf-α* production in liver of turbot [67]. Dietary BAs also downregulated the mRNA expression level of *tlr-8* in the present study. Toll-like receptor could mediate the activation of NF-*κ*B pathway in the conserved innate immunity [68]. These relative studies indicated that BAs would have a potential to mitigate the intestinal inflammatory response via suppressing TLR-NF-*κ*B pathway in fish fed diets containing higher lipid.

Claudins, occludin, and ZOs are the main tight junction proteins and are associated with the gut physical barrier functions [69]. In this study, dietary BAs upregulated mRNA expression level of *claudin-12* and *claudin-15*. The similar results of dietary BAs were certificated in tongue sole and largemouth bass, dietary BAs upregulated genes expression of physical barrier, including *claudin-2*, *claudin-4*, *claudin-7*, *occludin-2*, and *zo-1* [21, 70]. Thereby, dietary BAs can be used as a positive additive to enhance gut-tight junction functions. The physical barrier contributes to protecting intestinal health of aquatic animals [68]. This eel also showed that dietary BAs increased the intestinal fold height and lamina propria width of eel, which agreed with the studies in grass carp and largemouth bass [70]. Dietary BAs improved structural integrity in farmed fish. Based on the positive effects of dietary BAs on intestinal physical barrier and structural integrity, it is a reasonable speculation that dietary BAs could defense the intense from damage in farmed fish fed caused by HLDs.

Previous studies in snakehead [54] and leopard coral grouper (*Plectropomus leopardus*) [71] indicated that dietary BAs enhanced the lipase and trypsin activities. In addition, dietary BAs increased lipase and amylase activity whereas did not influence on protease in tongue sole [20]. Therefore, BAs were thought be a feed additive to improve feed utilization in these fish. However, in this study, dietary BAs did not change activities of trypsin and amylase, even decreased lipase activity. The complicated effect of dietary BAs on intestinal digestive enzyme was also proven in large yellow croaker, which BAs did not influence main digestive enzyme activities in intestine [72]. Dietary BAs improved feed utilization in this study and the previous study in large yellow croaker [72]. These different results indicated that the effect of dietary BAs on the interaction between digestive enzyme activities and feed utilization requires further research in the future.

## 5. Conclusion

BAs supplemented in HL diets (13%) of rice field eel could increase weight gain and reduce lipid deposition in liver, muscle, and whole body. Dietary BAs also improved intestinal antioxidant capability and showed positive effects on intestinal inflammatory response, oxidative stress, and physical barrier. Moreover, dietary BAs enhanced intestinal structural integrity. Thereby, dietary BAs (250 mg/kg) are recommended for use as an additive to enhance intestinal healthy in rice field eel fed with 13% lipid diet.

## Figures and Tables

**Table 1:** Formulation and proximate composition of the experimental diets.

Ingredients (%)	Diets			
	Con	HL	HLLB	HLHB
Peruvian steam fish meal^a^	35	35	35	35
Wheat gluten^a^	10	10	10	10
Soybean meal^a^	5.5	5.5	5.5	5.5
Wheat bran^a^	5.66	5.66	5.66	5.66
Chicken meal^a^	10	10	10	10
Squid paste^a^	3	3	3	3
Wheat starch^a^	20	20	20	20
Fish oil	1	3.9	3.9	3.9
Soybean oil	1	3.9	3.9	3.9
Ca(H_2_PO4)_2_	1.1	1.1	1.1	1.1
Choline chloride	0.7	0.7	0.7	0.7
Multidimensional^b^	0.15	0.15	0.15	0.15
Multi mineral^b^	1	1	1	1
Calcium propanoate	0.03	0.03	0.03	0.03
Ethoxyquin	0.01	0.01	0.01	0.01
Microcrystalline cellulose	5.85	0.05	0.025	0
Bile acids^c^	0	0	0.025	0.05
Total	100	100	100	100
Proximate analysis (%)				
Moisture	11.8	11.3	11.8	11.3
Ash	9.6	9.8	9.8	9.9
Crude protein	42.8	42.4	42.4	42.5
Crude lipid	6.97	13.10	13.14	13.18

^a^Composition of dry matter (%): peruvian steam fishmeal: crude protein 68.35, crude lipid 7.4; wheat gluten: crude protein 83.06, crude lipid 0.96; soybean meal: crude protein 44.2, crude lipid 1.9; wheat bran: crude protein 15.7, crude lipid 3.9; chicken meal: crude protein 65, crude lipid 10; squid paste: crude protein 30.1, crude lipid 20.72; wheat starch: crude protein 0.76, crude lipid 0.19. These ingredients were provided by Jiangxi Aohua Industrial (Nanchang, Jiangxi, China). ^b^Multidimensional and multimineral provided by MGO Ter Bio-Tech (Qingdao, Shandong, China). Vitamin and mineral premix composition (mg/kg diet): KCl, 200; KI (1%), 60; CoCl_2__6H_2_O (1%), 50; CuSO_4__5 H_2_O, 30; FeSO_4_ H_2_O, 400; ZnSO_4_ H_2_O, 400; MnSO_4__ H_2_O, 150; Na_2_SeO_3__5 H_2_O (1%), 65; MgSO_4__ H_2_O, 2000; zeolite power, 3645.85; VB1, 12; riboflavin, 12; VB6, 8; VB12, 0.05; VK3, 8; inositol, 100; pantothenic acid, 40; nicotinic acid, 50; folic acid, 5; biotin, 0.8; VA, 25; VD3, 5; VE, 50; VC, 100; ethoxyquin, 150; wheat meal, 2 434.15. ^c^Bile acids were bought from Shandong Longchang Animal Health Product Co., Ltd. (>95%, Jinan, China): hyodeoxycholic acid + hyocholic acid ≥77%, chenodeoxycholic acid ≥17%. Treatment groups: CON, control group; HL, high lipid diets group; HLLB, high lipid diets supplementing with 0.025% bile acid group; and HLHB, high lipid diets supplementing with 0.05% bile acid group.

**Table 2:** The primer of RT-PCR used in the experiment.

Gene	Forward sequences (5′⟶3′)		Reverse sequences (5′⟶3′)	Accession no.
*tnf-α*		TTTCAAGGAGGGCTGGTTCT	CTTGACCAGCGCATCACTGT	XM_020622780.1
*il-8*		ATGAGTCTTAGAGGTCTGGGT	ACAGTGAGGGCTAGGAGGG	XM_020597092.1
*tlr-8*		GAGGGCTACGTTAAGACTGGG	GACATTCCTCAGGCTTTGCC	XM_020596483.1
*il-10*		AATCCCTTTGATTTTGCC	GTGCCTTATCCTACAGTATGTG	XM_020593114.1
*nrf2*		CTTCAGACAGCGGTGACAGG	GCCTCATTCAGTTGGTGCTT	XM_020608174.1
*keap1*		AGCCTGGGTGCGATACGA	CAAGAAATGACTTTGGTGGG	XM_020597068.1
*claudin12*		TCACCTTCAATCGCAACG	ATGTCTGGCTCAGGCTTATCT	XM_020607277.1
*claudin15*		GGTCTCAGTGTCCTGGTACG	TGGTTTGATGGGACAACGGA	XM_020611334.1
*18s r RNA*		ATTTCCGACACGGAGAGG	CATGGGTTTAGGATACGCTC	XM_020605951.1

*tnf-α*, tumor necrosis factor alpha; *il-8*, interleukin-8; *tlr-8*, toll-like receptor 8; *il-10*, interleukin-8; *nrf2*, nuclear factor erythroid 2-related factor 2; *keapl*, kelch-like ECH associating protein 1; and 18s r RNA, 18s ribosomal RNA.

**Table 3:** Effects of dietary bile acids (BAs) on the growth performance of rice field eel (*Monopterus albus*) fed with HLDs.

Parameters	CON	HL	HLLB	HLHB	*P*_linear_
IBW (g)	17.04 ± 0.05	17.14 ± 0.02	17.14 ± 0.02	17.17 ± 0.06	ns
FBW (g)	48.88 ± 1.48	48.1 ± 0.76^a^	55.42 ± 0.75^c^	52.63 ± 0.01^b^	0.002
WGR (%)	186.33 ± 7.69	180.32 ± 4.25^a^	223.49 ± 4.88^c^	206.9 ± 1.94^b^	0.01
SGR (%/day)	1.88 ± 0.05	1.84 ± 0.03^a^	2.1 ± 0.03^c^	1.96 ± 0.04^b^	0.005
FCR	1.96 ± 0.05 ^*∗*^	1.75 ± 0.06^b^	1.5 ± 0.01^a^	1.86 ± 0.13^b^	0.02
HSI (%)	3.21 ± 0.12	3.78 ± 0.2	3.41 ± 0.31	3.84 ± 0.08	ns
VSI (%)	7.62 ± 0.3 ^*∗*^	8.74 ± 0.21	9.12 ± 0.25	9.15 ± 0.47	ns
CF (g/cm^3^)	0.22 ± 0 ^*∗*^	0.21 ± 0^a^	0.23 ± 0^b^	0.24 ± 0.01^c^	0.017

*Note*: Each data point represents the mean of four replicates (*n* = 4, mean ± SEM).  ^*∗*^Means *P* < 0.05 between the CON and the HL groups. Means in each row sharing the different superscripts are significantly different (*P* < 0.05) among the HL, HLLB, and HLHB groups. *P*_linear_ means statistical results of orthogonal polynomial contrasts. ns, no significant. The following variables were calculated: survival rate (SR, %) = Nt × 100∕No. weight gain rate (WGR, %) = (Wt − Wo) × 100∕Wo. Specific growth rate (SGR, %d − 1) = (LnWt – LnWo) × 100∕t. Feed conversion rate (FCR, %) = 100 × (total amount of the feed consumed∕(Wt − Wo)). Condition factor (CF, g/cm^3^) = 100 × (Wt − Wo)∕(body length)^3^. Hepatosomatic index (HSI, %) = 100 × (liver weight∕Wt). Viscerosomatic index (VSI, %) = 100 × (visceral weight∕Wt). Where Wt and Wo were final body weight (FBW, g) and initial body weight (IBW, g), Nt and No were final and initial number of fish, respectively, and it was the duration of experimental days. Treatment groups: CON, control group; HL, high lipid diets group; HLLB, high lipid diets supplementing with 0.025% bile acid group; and HLHB, high lipid diets supplementing with 0.05% bile acid group.

## Data Availability

Data supporting this reasearch article are available on request.
